# Which Individuals To Choose To Update the Reference Population? Minimizing the Loss of Genetic Diversity in Animal Genomic Selection Programs

**DOI:** 10.1534/g3.117.1117

**Published:** 2017-11-13

**Authors:** Sonia E. Eynard, Pascal Croiseau, Denis Laloë, Sebastien Fritz, Mario P. L. Calus, Gwendal Restoux

**Affiliations:** * Génétique Animale et Biologie Intégrative (GABI), Institut National de la Recherche Agronomique (INRA), AgroParisTech, Université Paris-Saclay, 78350 Jouy en Josas, France; † Animal Breeding and Genomics Centre, Wageningen University & Research, 6700 AH Wageningen, The Netherlands; ‡ Centre for Genetic Resources, Wageningen University & Research, the Netherlands, 6700 AA Wageningen, The Netherlands; § Allice, 75595 Paris Cedex 12, France

**Keywords:** genomic selection, genetic diversity, reference population, optimal contribution, GenPred, Shared data resources

## Abstract

Genomic selection (GS) is commonly used in livestock and increasingly in plant breeding. Relying on phenotypes and genotypes of a reference population, GS allows performance prediction for young individuals having only genotypes. This is expected to achieve fast high genetic gain but with a potential loss of genetic diversity. Existing methods to conserve genetic diversity depend mostly on the choice of the breeding individuals. In this study, we propose a modification of the reference population composition to mitigate diversity loss. Since the high cost of phenotyping is the limiting factor for GS, our findings are of major economic interest. This study aims to answer the following questions: how would decisions on the reference population affect the breeding population, and how to best select individuals to update the reference population and balance maximizing genetic gain and minimizing loss of genetic diversity? We investigated three updating strategies for the reference population: random, truncation, and optimal contribution (OC) strategies. OC maximizes genetic merit for a fixed loss of genetic diversity. A French Montbéliarde dairy cattle population with 50K SNP chip genotypes and simulations over 10 generations were used to compare these different strategies using milk production as the trait of interest. Candidates were selected to update the reference population. Prediction bias and both genetic merit and diversity were measured. Changes in the reference population composition slightly affected the breeding population. Optimal contribution strategy appeared to be an acceptable compromise to maintain both genetic gain and diversity in the reference and the breeding populations.

The development of genomic selection (GS), as described by Meuwissen *et al.* (2001), is the most important recent innovation in animal breeding. In livestock breeding, GS comprises the estimation of genomic estimated breeding values (GEBVs) and the actual selection of individuals with only genotypes available, *e.g.*, young individuals that are candidates for selection, based on these GEBVs (Supplemental Material, Figure S1). A reference population, composed of individuals with known phenotypes and genotypes based on many markers across the genome, is used to set up prediction equations and infer GEBVs of selection candidates. The main advantages of GS, compared to the traditional methods based on phenotype and pedigree, are that generation intervals are reduced since phenotypes of mature progenies are no longer needed to perform genetic evaluation. Second, selection can still be performed with the same accuracy as classical selection. Lastly, it allows selection for new traits that are difficult and costly to record (Meuwissen *et al.* 2001; Calus and Veerkamp 2011). Despite the confirmed advantages, most of the knowledge on the long-term impact of GS is based on simulation studies [*e.g.*, Colleau *et al.* (2009), Jannink (2010), Bastiaansen *et al.* (2012), and Clark *et al.* (2013)] and many questions remain concerning its use. In particular about the design of the reference population: how many individuals are needed (Pszczola *et al.* 2011; Khatkar *et al.* 2012; Pryce and Daetwyler 2012), how often should marker effects be reestimated (Calus 2010; Heslot *et al.* 2013), how closely related should individuals in the reference population be to the selection candidates (Pszczola *et al.* 2012a; Meuwissen *et al.* 2013), and which individuals should be used to update the reference population (Rincent *et al.* 2012; Isidro *et al.* 2015)?

Many livestock breeds have high inbreeding rates and low genetic diversity as a result of intensive selection (Leroy *et al.* 2011). Limited genetic diversity restricts the potential long-term genetic gain of the populations (Li *et al.* 2008; Goddard 2009; Jannink 2010; Engelsma *et al.* 2012; Liu 2013; Henryon *et al.* 2014) and reduces their ability to respond to new challenges (Toro *et al.* 2009; Allendorf *et al.* 2010; Stock and Reents 2013; Bruford *et al.* 2015). To allow for long-term maintenance, individuals representing the overall population’s diversity need to be used for breeding (Rincent *et al.* 2012; Heslot *et al.* 2013; Isidro *et al.* 2015). Different strategies have been previously suggested: (1) limiting the number of offspring per male to avoid the sire “star system” (Danchin-Burge *et al.* 2012; Boichard *et al.* 2015), (2) distinguishing individuals according to the marker variation they carry and giving extra weight to the low-frequency favorable markers (Jannink 2010), or (3) choose individuals to represent the highest overall population diversity (Meuwissen 1997; Rincent *et al.* 2012; Heslot *et al.* 2013). One of the available methods developed for such a goal is the optimal contribution (OC) strategy, as defined by Meuwissen (1997). The OC strategy can be used to simultaneously conserve genetic diversity and achieve genetic gain by minimizing the relationships between the individuals (Engelsma *et al.* 2011; Sonesson *et al.* 2012; Clark *et al.* 2013; de Cara *et al.* 2013; Eynard *et al.* 2016). The effectiveness of these methods relies on the final choice of the breeding individuals. In the case of dairy cattle, such strategies to conserve overall population genetic diversity may be insufficiently used in the context of competitive economical markets promoting the use of elite reproducers. Methods implicitly driving selection toward both genetic gain and the maintenance of genetic diversity may be an alternative. With the design of the reference population there is the potential to modify the breeding population by changing the genetic evaluation.

In this study, we addressed the following question: how does one choose individuals to update the reference population of a GS scheme in order to balance genetic gain and genetic diversity? We anticipate that changes in the composition of the reference population will be associated with changes in the breeding population due to adjustments of the prediction equations for GS. To test this hypothesis, we compared three different strategies (random, truncation, and OC) to select individuals for the update of the reference population. Using a real data set of French dairy cattle (Montbéliarde), we focused on the effect of updating strategies on the population of selected candidates. Using simulations, we inferred the long-term effect of these updating strategies on the breeding population. For both real and simulated data sets, updating strategies were evaluated in terms of genetic merit, genetic diversity, and performances of GS.

## Materials and Methods

### Real data set

A population of 14,052 individuals from the French Montbéliarde dairy cattle breed, 2459 males and 11,593 females, born between 1969 and 2011 was available for the analysis. The complete pedigree record contained 50,852 individuals born from the 1940s until 2011. All individuals had, at the very least, complete pedigree records for their parental generation with a maximum of seven complete generations. The generation equivalents [sum of the proportion of known ancestors in all available generations (Maignel *et al.* 1996)] ranged from two to nine. For all individuals 50K SNP genotypes were available. Males were genotyped using the BovineSNP50 v2 BeadChip (Illumina) and females were genotyped using the 10K SNP chip (Illumina) and subsequently imputed, by Hoze *et al.* (2013), to the BovineSNP50 v2 BeadChip using the BEAGLE software (Browning and Browning 2007). The software DAGPHASE (Druet and Georges 2010) was used for phasing. Subsequent quality control steps were required for each SNP: (i) a minimum call rate > 90%, (ii) nondeparture from Hardy–Weinberg equilibrium (p-values < 10^−4^), and (iii) MAF > 1%, to minimize potential genotyping errors. The final genotype data comprised 43,801 markers genotyped on the 29 autosomes. In this study, we focused on milk yield having heritability of 0.3, a genetic variance of 423,390 kg^2^, and a residual variance of 987,910 kg^2^. Milk yield was measured as the corrected milk yield for the females with, on average, 1.66 records per female. For the progeny-tested males, milk yield was measured as daughter yield deviation, reflecting the average milk yield of their daughters adjusted for fixed and nongenetic random effects and the additive genetic value of their dam (Mrode and Swanson 2004). Weights used for male records were defined as effective daughters’ contribution (Fikse and Banos 2001) and were on average 26.21. The data set was divided into three groups according to individuals’ birth years. The first group included 5969 individuals (2325 males and 3644 females) born between 1969 and 2007 and was used as the initial reference population for GS (*A*_1_). The second group included 3791 individuals (134 males and 3657 females) born in 2008 and 2009, and those individuals were considered to be available to be added to the updated reference population (*A*_2_). The third group included 4292 individuals (all females) born in 2010 and 2011, and was used for validation of the GS (*V*) (Figure S2).

### Simulation process

**Figure 1:**
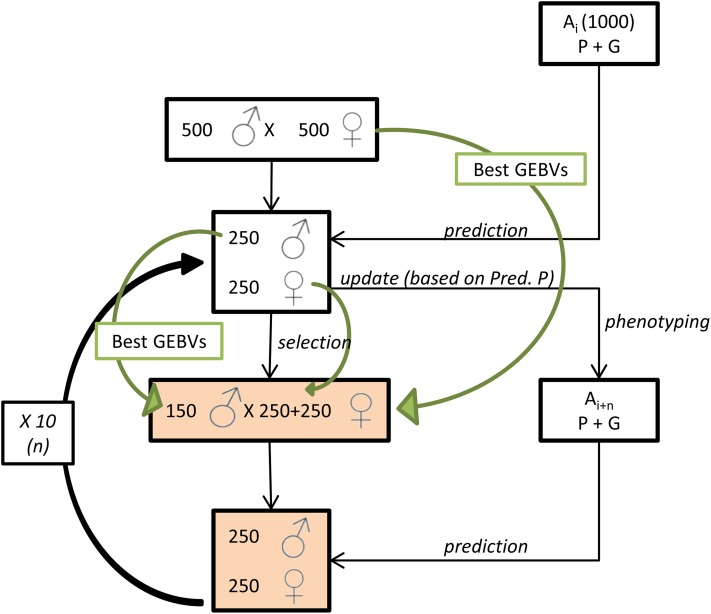
Simulation design. This figure represents the scheme used for simulations. The highlighted boxes represent the population under consideration. The green arrows inform on the selection decision. G, genotype; GEBV, genomic estimated breeding value; P, phenotype; Pred. P, predicted phenotype.

We simulated a population with characteristics similar to a domestic cattle population and a trait similar to milk yield. An ancestral population of 1000 males and 1000 females that had undergone selection based on estimated breeding values (EBVs) estimated from a best linear unbiased prediction (BLUP) method was used as the starting point of our simulations. Next, 10 more generations of selection and breeding were simulated. In every generation, the 150 males and 500 females from the previous generations with the highest GEBVs were selected to produce the next generation *n* + 1 (a selection rate of 0.6 for the males, of 1 for the females from the generation *n*, and of 0.5 for the females from the generation *n* − 1). Males could reproduce for one generation while females could produce offspring in multiple generations assuming that their GEBVs were high enough. We assumed that selection excluded them from the population after 2 yr. Each female produced one offspring per generation and the sex ratio in the offspring generation was 0.5 (Figure 1). The simulated design is simpler than what occurs in a real breeding scheme. Simulations were performed using QMSim (Sargolzaei and Schenkel 2009). Details of the simulation process are provided in Supplemental Material (File S1).

### Genomic best linear unbiased prediction (GBLUP)

To investigate the impact of an update to the reference population on GS in terms of subsequent predicted GEBVs, we used both real and simulated data sets. The real data set allowed us to study the impact of reference population updating strategies on the choice of breeding individuals for the next generation only. Simulations were used to study the impact on the breeding population over multiple generations. GEBVs were predicted by a GBLUP model fitted with GS3 software (Legarra *et al.* 2011). For the GBLUP model (Croiseau *et al.* 2011): (i) the estimated relationship matrix was calculated according to the VanRaden (2008) equation $G=ZZ^{\prime }/2\sum_{i=1}^{m}p_{i}\left(1-p_{i}\right)$, where **Z** is the genotype matrix and $p_{i}$ the allele frequency of marker *i*, (ii) the variance components for this trait were the ones used in the routine evaluation in France and were fixed in the model, and (iii) only random effects were fitted as the phenotypes used were already corrected for fixed effects and nongenetic random effects.

### Reference population update

Three updating strategies were compared: (1) selection at random (Random) repeated 100 independent times, (2) truncation selection based on the highest GEBVs (Sel), and (3) selection to simultaneously maximize the genetic diversity and the genetic merit of the group of selected individuals (SelDiv) using the OC strategy and the Gencont program (Meuwissen 1997). The genetic merit of a set of selected individuals is the average breeding value (BV) of the selected individuals. The rate of inbreeding (∆F) between the current and next generation is estimated from the average genomic relationships of selected individuals. The OC method identifies a set of individuals with maximum genetic merit with the restriction that the expected rate of inbreeding is no > 1%, as recommended by the FAO (1998). If the given constraint of 1% cannot be met because of population structure, then the choice of individuals is made to minimize the rate of inbreeding and genetic merit is effectively not considered. The SelDiv strategy used genomic relationships, computed as similarities that count the number of identical alleles, averaged across loci between two individuals (Nejati-Javaremi *et al.* 1997; Eding and Meuwissen 2001):

$$G_{jk}=\frac{2}{N}\underset{i}{\sum }(x_{ij}-1)(x_{ik}-1)$$

where *N* is the number of markers and $G_{jk}$ is the estimated relationship between individual *j* and *k* across all markers. At each marker, *i*, $x_{i}$ is the individual variant genotype coded as 0, 1, or 2. Note that computing these relationships using the methods described by VanRaden (2008) and Yang *et al.* (2010), assuming allele frequencies of 0.5 for all loci, yields exactly the same result. This relationship matrix has been shown to reduce the loss of overall genetic diversity better than other relationship matrices when applying the OC strategy (Eynard *et al.* 2016).

#### Update of reference population in real data sets:

The initial reference population (*A*_1_) was used to predict GEBVs of the individuals in the candidates’ population (*A*_2_). Using these GEBVs and the relationships between individuals in *A*_1_ and *A*_2_, we selected subgroups of individuals to build updated reference populations (*A*_1+2_) For all strategies (Random, Sel, and SelDiv), the initial reference population (*A*_1_) of 5969 individuals was updated with 100, 200, 500, 1000, or 2000 new individuals, which represented adding ∼1.5, 3, 8, 15, and 30% to the initial reference population, respectively. The updated reference populations (*A*_1+2_) were used to predict GEBVs of the candidates’ group *V*. Based on their GEBVs, the top 100 individuals from *V* were selected as breeding populations, *V_sel_*. A detailed review of all results is available in Table S1 in File S2.

#### Update of reference population in simulated data sets:

The initial reference population (*A*_1_) consisted of 1000 males from the ancestral individuals and was updated every generation by adding 150 individuals, males and/or females, selected based on one of the proposed strategies (Random, Sel, and SelDiv). The size of the reference population therefore rose from 1000 in the first generation to 2350 individuals in the 10th generation. In each generation, the reference population was updated based on GEBVs from the candidates’ population, and subsequently used for prediction of GEBVs of the simulated offspring. Therefore, individuals in the reference population could be included as part of the breeding population provided that they had been selected for breeding based on their GEBVs. The whole simulation and updating process was replicated 50 times for each strategy.

### Evaluation of updating strategies

To compare the different updating strategies, several parameters were evaluated for the selected candidates’ population (*V_sel_*, top 100 individuals) in the real data set and for the breeding population in the simulated data set. Those parameters included: (i) the response to selection, (ii) the genetic diversity, (iii) prediction bias, and (iv) the effective population size of the reference population. Response to selection was measured as the change in average BV. Genetic diversity was measured as: (i) observed heterozygosity and (ii) the inbreeding coefficient obtained from pedigree following the Sargolzaei *et al.* (2005) algorithm. The bias of GEBV was measured by the absolute standardized prediction errors for the BV as follows:

$${\text{Bias}}_{k}=\left|\frac{{\text{GEBV}}_{k}-{\text{BV}}_{k}}{\sigma_{{\text{G}}_{i}}}\right|,$$

where ${\text{GEBV}}_{k}$ is the GEBV of the individual *k*, ${\text{BV}}_{k}$ is the BV (based on multiple records in the real data set or given by the simulations in the form of a true BV) of the individual *k*, and $\sigma_{\text{G}}$ is the true BV SD of the population under scrutiny *i*. The effective population size of the reference population, *N_e_*, was also estimated following the classical formula derived from the inbreeding coefficient definition (Falconer and Mackay 1996):

$$N_{e}=\;\frac{1}{2*f_{t}}$$,

with *f_t_* representing the mean inbreeding coefficient of the population in the *t*th generation.

The effects of the different updating strategies on BV, heterozygosity, inbreeding, and prediction bias were tested using linear models implemented in R and the *lme4* package (Bates *et al.* 2015; R Core Team 2016), considering the random strategy as the null hypothesis distribution. When dealing with heterozygosity or inbreeding, an arcsine-square root transformation was applied to ensure the applicability of linear models. The effects of strategy and the size of the update were tested using a type II ANOVA [R package *car* (Fox and Weisberg 2011)]. Coefficients of change throughout generations were compared using least square means for qualitative variables and least square trends to compare regression slopes for quantitative variables [R package *lsmeans* (Lenth 2016)].

For the real data set, linear models were applied on the candidates’ populations as follows,

$$Y_{ijk}=\mu +{\text{strategy}}_{i}+{\text{update size}}_{j}+{\left(\text{strategy}\times \text{update size}\right)}_{ij}+\beta_{1}{\left(\frac{N_{e}}{N}\right)}_{ij}+\varepsilon_{ijk},$$

where $Y_{ijk}$ is the variable measured on individual *k*, for strategy *i* (Random, Sel, or SelDiv), when adding ${\text{update size}}_{j},$ number of individuals added to the reference population, fitted here as a qualitative effect (100, 200, 500, 1000, or 2000). $\beta_{1}$ is the regression coefficient on the ratio $N_{e}/N\;$of the reference population (with *N* the census population size) and $\varepsilon_{ijk}$is the Gaussian residual. For simulated data sets, we focused on the breeding and offspring populations using the following mixed effects models,

$$Y_{ilk}=\mu +{\text{strategy}}_{i}+\beta_{2,i}\left({\text{generation}}_{l}\right)+\alpha_{i}\left({\text{generation}}_{l}\right)*\left({\text{strategy}}_{i}\right)+\beta_{1}{\left(\frac{N_{e}}{N}\right)}_{il}+Sim_{l}\;+\varepsilon_{ilk},$$

$$Sim_{l}∼N(\mu =0,\sigma_{sim}^{2}),$$

where $Y_{ilk}$ is the variable measured on individual *k*, for the strategy *i*, in generation *l* of simulation, $\beta_{2,i}$ the regression coefficient on the generation number for strategy *i*, $\alpha_{i}$ is the interaction effect of method with generation, and $Sim_{l}$ was the random effect of the simulation where $\sigma_{sim}^{2}$ represented the data variability among simulation replicates and $\varepsilon_{ijk}\;$ the gaussian residuals. The ratio $N_{e}/N$ of the reference population was used in the model to account for the effect of the change in reference population size through time while accounting for a parallel growth of census population size. This allows one to distinguish between the increases in size over time from the cumulative effect due to consecutive population changes over the 10 generations.

### Data availability

Genetic information (in the form of a **G**-matrix), pedigree (for the individuals under scrutiny), and BV for the trait of interest are available for the real data set, as well as the script allowing the production of the simulated data sets and documents describing each file for real and simulated data sets on the following depository: doi.org/10.5281/zenodo.1066566.

## Results

### Effect of updating strategy on selected candidates (real data sets)

#### Genetic merit of the selected candidates:

Individual BVs in *V_sel_* exhibited large variability and ranged from 461 to 5674. Average BV of *V_sel_* populations, across all combinations of strategies and the size of updates, ranged from 3153.56 to 3185.63 (± 5.21), thus revealing limited variation in genetic gain between different strategies to update the reference population. Even though none of these differences were significant, genetic merit tended to increase when increasing the size of the group used to update the reference population.

#### Genetic diversity of the selected candidates:

Individuals’ inbreeding ranged from 0.02 to 0.11. Over all combinations of strategies and size of updates, per *V_sel_*, the inbreeding coefficients were all on average 0.05 (± 1.14 × 10^−4^) and not significantly different from each other. Individuals’ heterozygosity ranged from 0.28 to 0.33, and average populations’ heterozygosities were all close to the mean value of 0.31 (± 5.65 × 10^−5^) and not significantly different across scenarios.

#### Precision of GEBV prediction procedure:

The prediction bias of GEBVs of the full candidates’ population, *V*, ranged from 0.00 to 7.73, indicating substantial disparity in how well individuals’ GEBVs are predicted. Across all combinations of strategies and size of updates, average absolute bias of GEBV ranged from 1.05 to 1.08 (± 0.01) without any significant difference among them (Table 1).

**Table 1:** Descriptive statistics of the four variables analyzed at group level, for the different strategies and sizes of update in the real data set

		Breeding Value		Absolute Prediction Bias		Inbreeding		Observed Heterozygosity	
Update Size	Selection Strategy	Average	95% C.I.	Average	95% C.I.	Average	95% C.I.	Average	95% C.I.
100	Sel	3182.63		1.08		5.06 × 10^−2^		3.07 × 10^−1^	
	SelDiv	3158.71		1.08		5.06 × 10^−2^		3.07 × 10^−1^	
	Random	3159.69	3159.30 to 3160.08	1.08	1.08 to 1.08	5.05 × 10^−2^	5.04 × 10^−2^ to 5.05 × 10^−2^	3.07 × 10^−1^	3.07 × 10^−1^ to 3.07 × 10^−1^
200	Sel	3163.79		1.07		5.03 × 10^−2^		3.07 × 10^−1^	
	SelDiv	3159.21		1.08		5.03 × 10^−2^		3.07 × 10^−1^	
	Random	3161.05	3160.43 to 3161.67	1.08	1.08 to 1.08	5.04 × 10^−2^	5.04 × 10^−2^ to 5.04 × 10^−2^	3.07 × 10^−1^	3.07 × 10^−1^ to 3.07 × 10^−1^
500	Sel	3181.93		1.06		5.03 × 10^−2^		3.08 × 10^−1^	
	SelDiv	3165.91		1.07		5.04 × 10^−2^		3.07 × 10^−1^	
	Random	3162.64	3161.83 to 3163.45	1.07	1.07 to 1.07	5.04 × 10^−2^	5.04 × 10^−2^ to 5.05 × 10^−2^	3.07 × 10^−1^	3.07 × 10^−1^ to 3.07 × 10^−1^
1000	Sel	3181.93		1.05		5.03 × 10^−2^		3.08 × 10^−1^	
	SelDiv	3168.00		1.06		5.03 × 10^−2^		3.07 × 10^−1^	
	Random	3165.02	3163.84 to 3166.19	1.06	1.06 to 1.06	5.04 × 10^−2^	5.04 × 10^−2^ to 5.05 × 10^−2^	3.07 × 10^−1^	3.07 × 10^−1^ to 3.07 × 10^−1^
2000	Sel	3178.40		1.05		5.03 × 10^−2^		3.07 × 10^−1^	
	SelDiv	3163.26		1.06		5.03 × 10^−2^		3.07 × 10^−1^	
	Random	3166.19	3165.13 to 3167.24	1.06	1.06 to 1.06	5.04 × 10^−2^	5.04 × 10^−2^ to 5.04 × 10^−2^	3.07 × 10^−1^	3.07 × 10^−1^ to 3.07 × 10^−1^

Random, selection at random repeated 100 independent times; Sel, truncation selection based on the highest genomic estimated breeding values; SelDiv, selection to simultaneously maximize the genetic diversity and the genetic merit of the group of selected individuals using the optimal contribution strategy and the Gencont program.

Overall, no significant differences could be observed between the three tested strategies when considering the top 100 candidates for selection.

### Long-term effect of updating strategy on breeding population (simulated data sets)

#### Genetic merit of the breeding population:

**Figure 2:**
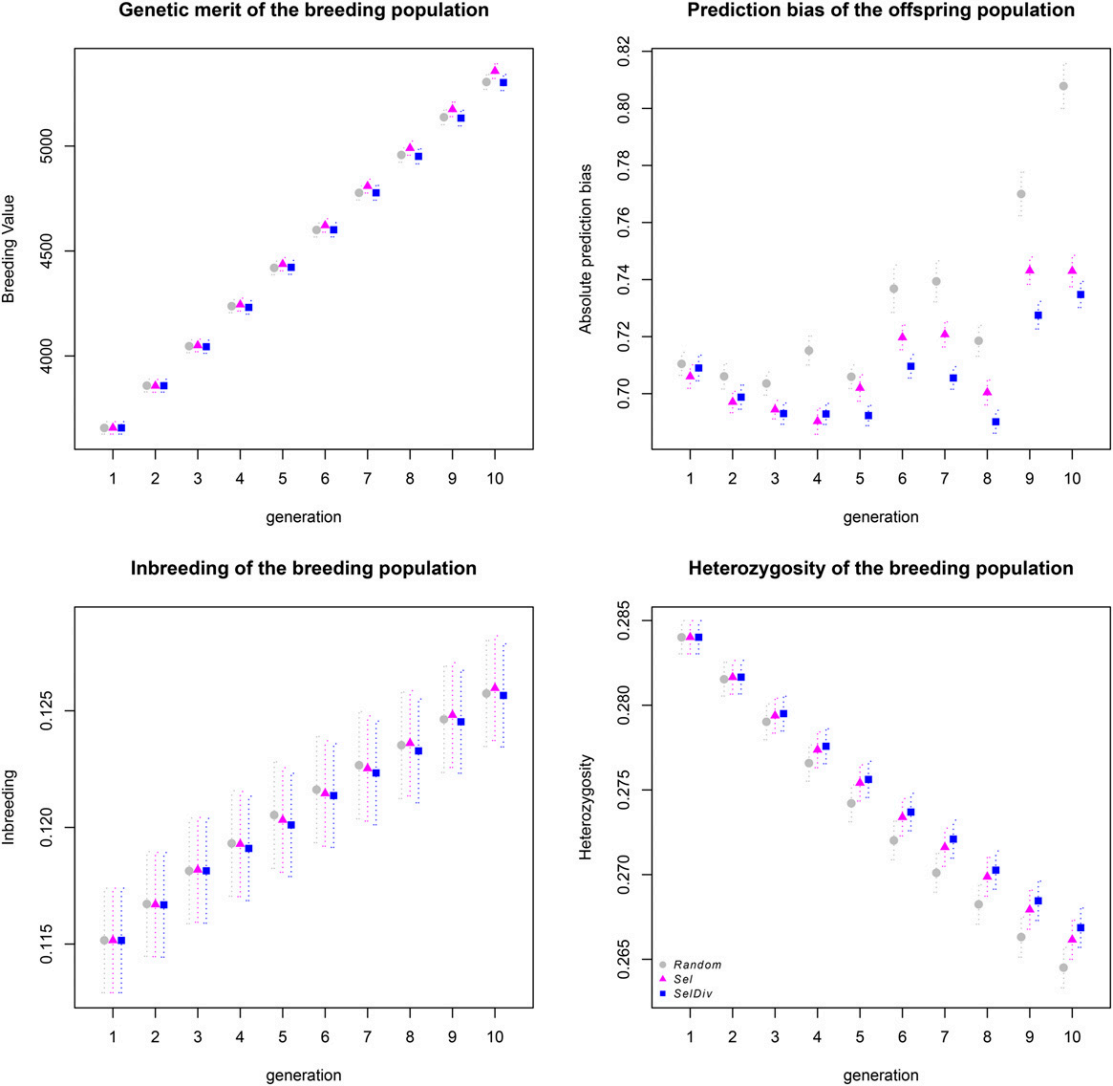
Evolution of genetic merit, performance of genomic selection, and genetic diversity over 10 generations of simulations for different update strategies. The four plots represent the average genetic merit of the breeding populations (top left), average prediction bias of genomic estimated breeding values of the offspring populations (top right), the average inbreeding (bottom left), and the average heterozygosity (bottom right) of the breeding populations over 10 generations of selection. For the three update strategies Random (gray circle), Sel (magenta triangles), and SelDiv (blue squares), the average values and SE are represented. Random, selection at random repeated 100 independent times; Sel, truncation selection based on the highest genomic estimated breeding values; SelDiv, selection to simultaneously maximize the genetic diversity and the genetic merit of the group of selected individuals using the optimal contribution strategy and the Gencont program.

The average BV of the breeding population always increased from one generation to the next. Despite the fact that strategy significantly affected the realized genetic merit (all p-values < 10^−5^, Table S3 in File S2), the actual differences between the Sel, SelDiv, and Random strategies were very modest (Figure 2, Table S2 in File S2, and Table 2).

**Table 2:** Trends of changes throughout the 10 generations of simulation for each of the three updating strategies and four variables

	Generation Trend	SE	95% C.I.
Breeding value			
Sel	173.77	7.07 × 10^−1^	172.38 to 175.15
SelDiv	167.69	7.05 × 10^−1^	166.30 to 169.07
Random	167.93	7.07 × 10^−1^	166.55 to 169.32
Prediction bias			
Sel	4.03 × 10^−2^	1.30 × 10^−3^	3.77 × 10^−2^ to 4.28 × 10^−2^
SelDiv	3.40 × 10^−2^	1.29 × 10^−3^	3.14 × 10^−2^ to 3.65 × 10^−2^
Random	6.57 × 10^−2^	1.30 × 10^−3^	6.31 × 10^−2^ to 6.82 × 10^−2^
Inbreeding			
Sel	1.19 × 10^−3^	2.96 × 10^−5^	1.13 × 10^−3^ to 1.24 × 10^−3^
SelDiv	1.14 × 10^−3^	2.96 × 10^−5^	1.08 × 10^−3^ to 1.20 × 10^−3^
Random	1.16 × 10^−3^	2.96 × 10^−5^	1.10 × 10^−3^ to 1.22 × 10^−3^
Observed heterozygosity			
Sel	−2.10 × 10^−3^	2.17 × 10^−5^	−2.14 × 10^−3^ to −2.06 × 10^−3^
SelDiv	−2.02 × 10^−3^	2.16 × 10^−5^	−2.06 × 10^−3^ to −1.97 × 10^−3^
Random	−2.33 × 10^−3^	2.17 × 10^−5^	−2.38 × 10^−3^ to −2.29 × 10^−3^

Random, selection at random repeated 100 independent times; Sel, truncation selection based on the highest genomic estimated breeding values; SelDiv, selection to simultaneously maximize the genetic diversity and the genetic merit of the group of selected individuals using the optimal contribution strategy and the Gencont program.

#### Genetic diversity of the breeding population:

Whatever the strategy, the inbreeding coefficient increased from one generation to the next. Despite large SE (Figure 2), the increase in inbreeding coefficients throughout the 10 generations appeared to be significantly slower for SelDiv than for the two other strategies (Table 2). Inbreeding level was significantly associated with both generation number and $N_{e}/N$ (p-values < 10^−16^, Table S3 in File S2). Both an increase in generation number and a decrease in $N_{e}/N$ was associated with an increase of the average population inbreeding. After the fourth generation, the SelDiv strategy resulted in higher heterozygosity than the Sel or Random strategies (Figure 2) due to a slower decrease over generations (Table 2). All the parameters—strategy (p-value = 1.12 × 10^−2^), $N_{e}/N$ (p-value = 1.26 × 10^−6^), generation number, and the interaction between strategy and generation (both with p-values < 10^−16^)—significantly affected the heterozygosity (Table S3 in File S2). The effect of $N_{e}/N$ was positive; an increase in $N_{e}/N$ caused an increase in average heterozygosity of the population. Average heterozygosity decreased from one generation to the next faster for the Random and Sel strategies than for SelDiv.

#### Precision of GEBV prediction procedure:

For all generations, on average the Sel strategy, and even more the SelDiv strategy, resulted in lower prediction bias of the offspring’s GEBVs than the Random strategy (Table S2 in File S2). The parameters strategy, generation number, interaction between strategy and generation, and $N_{e}/N$ significantly affected prediction bias, with p-values < 10^−10^ (Table S3 in File S2). The Random, Sel, and SelDiv strategies were significantly different from each other (Table 2). A shift was observed at the fourth generation, with the Random strategy having the largest bias, whereas the SelDiv strategy had the lowest bias (Figure 2). Despite the apparently chaotic behavior of this variable, prediction bias tended to increase over time faster for the Random and Sel strategies than SelDiv. The small effect of $N_{e}/N$ on the prediction bias is presumably due to the decline in relationships between reference and candidate populations through time, as a result of the constant addition of new individuals without the removal of older ones.

To summarize, the results above show that different strategies to update the reference population have a significant, but small, impact on the breeding population. The SelDi*v* strategy resulted in slightly higher genetic diversity in the breeding population accompanied by a minor impact on the genetic gain and lower long-term prediction bias.

## Discussion

In this study, we compared the impact of different strategies to update the reference population in a GS framework on the genetic merit and diversity of the resulting breeding population. Optimizing the updating strategy is especially important in artificial selection based on the genotypes of individuals at an early age. This is because phenotyping is the limiting factor due to the time and money investment for the rearing of the individuals (Colleau *et al.* 2009; Konig *et al.* 2009). It is also relevant when both phenotypes and genotypes are available, but only a fraction can be included in the reference population, for example, when designing a core collection in plant breeding (Rincent *et al.* 2012; Isidro *et al.* 2015). In GS, reference population design and breeding decisions are linked through GEBVs of selection candidates. Our hypothesis was that the choice of individuals in building the reference population might impact the GEBVs of selection candidates and, consequently, the breeding population, both in terms of genetic gain and diversity.

### Long-term impact of updating strategy on the breeding population

Analysis based on a single generation in the real data set did not show significant differences between the three proposed updating strategies; however, analysis based on simulated data sets over 10 generations did show significant effects of the updating strategy on the breeding populations over time. A small beneficial response of the truncation strategy was observed for genetic merit, while the OC strategy performed best at conserving genetic diversity.

A recent study by De Beukelaer *et al.* (2017) focused on the similar question of how to balance genetic gain and genetic diversity conservation in populations under selection. The authors used simulations to compare established selection strategies: GS including OC (GOCS) and GS weighting for rare alleles (GSW) for long-term genetic diversity conservation in plant breeding. Even though both GOCS and GSW outperformed GS for long-term genetic gain, they were not successful in controlling inbreeding rate and loss of rare variants in the breeding population. These authors proposed two new strategies combining an index-based method and expected heterozygosity or rare allele frequencies as alternatives outperforming GS, GOCS, and GSW in balancing genetic gain and diversity. These methods require further investigation to confirm their benefits in practice but could be of potential interest to answer the questions we raised in this study.

Approaches proposed in plant breeding to design reference populations representing population structure and diversity (Laloe 1993; Rincent *et al.* 2012; Isidro *et al.* 2015; Bartholomé *et al.* 2016) could also be alternatives in the context of animal breeding. In fact, the current concerns of how to best design reference populations by targeting only relevant individuals is also now of interest in animal breeding due to the increasing availability of individual information both for phenotypes and genotypes. The data on livestock reference populations are now far more comprehensive and should enable choices regarding which individuals should be present in reference populations to take place. Therefore, methods used in plant breeding, mostly to design core collections, may be of interest to animal breeders.

### Potential implications for animal breeding

In practice, breeding decisions are mainly based on the genetic merit of individuals. This is because breeders’ incomes come from production. This phenomenon is putting small breeds in a difficult situation, in a market mostly dominated by mainstream breeds, because of their limited population size, high inbreeding rates, and lower fitness potential (Toro *et al.* 2009; Allendorf *et al.* 2010; Pryce and Daetwyler 2012). Livestock breeding has to balance the conservation of genetic diversity against genetic gain. Within GS, the adoption of alternative selection strategies, such as OC, is not common in practice. Acting on the reference population to directly mitigate the loss of genetic diversity of the breeding population, while only marginally affecting the genetic gain over generations, is a promising way to incorporate genetic diversity into breeding programs. Indeed, current methods to cope with the loss of genetic diversity mainly deal with the choice of which individuals to keep in the breeding population according to their estimated performances. On the one hand, direct selection of breeding individuals has the advantage of having a strong impact on both the level of genetic diversity and genetic gain, depending on the method used. On the other hand, it relies on the choice of the breeders and is thus not systematic. Here, we propose an integrated method to cope with genetic diversity at the genetic evaluation level, making it systematically incorporated. Thus, even if its impact on the conservation of genetic diversity is weaker than direct choices in the short-term, it potentially has a more consistent impact on a long-term basis. We expect that in the ideal case of operating on both the reference and breeding population, the effect observed would be further amplified and thus have an important impact on genetic diversity conservation.

### Limitations and perspectives of the study

The 50K SNP chip is routinely used in GS because of its low cost and fair performance for genetic gain. Several studies cautioned that the accuracy of prediction in GS when using whole-genome sequencing (WGS) was, at best, marginally higher than of the SNP chips (van Binsbergen *et al.* 2015; Calus *et al.* 2016; Lund *et al.* 2016; van den Berg *et al.* 2016; Ni *et al.* 2017). Still, we can hypothesize that using WGS or genotypes of higher density could favor larger differences in genetic diversity conserved between the described scenarios. This may be especially true in the case of rare variant sites, since they are underrepresented in the SNP chip compared to WGS (Eynard *et al.* 2015, 2016). Using WGS could enable the OC strategy to better conserve rare variants during updates of the reference population.

Prediction bias appeared to be smaller in the case of the OC strategy compared to the other two strategies. Increasing the genetic diversity of the reference population increased our representation of the overall population diversity and seemed to lead to slightly more accurate overall prediction. This is potentially thanks to an improved prediction of “outsider” variants. Additionally, particular attention should be paid to how many and which individuals should be removed. In fact, bias was first reduced by the addition of specifically selected individuals (Pszczola *et al.* 2012b). However, after some generations, adding individuals elevated the prediction bias. This is probably due to the lack of a relationship between the old individuals of the reference population and the candidates for selection. There is a need for further investigation in order to give recommendations as to the best updating strategy for reference populations, accounting for the addition and removal of individuals. Finally, our study is based on milk production, a trait of major interest in current livestock, with moderate heritability (0.3) that is similar to the those of composite index traits that represent the entire breeding goal. An important question is how results would change when the heritability is lower, because GS is especially appealing for low-heritability traits. Using a lower heritability, while leaving the reference population size unchanged, would have yielded lower prediction accuracies and also smaller differences between scenarios. A lower accuracy means that more emphasis is put on information from relatives, such that the EBV of relatives becomes more correlated and thus selected individuals are more likely to be related. This would result in conserving less genetic diversity and more inbreeding depression. Increasing the size of the reference population could counteract these effects of a low-heritability trait, because it would increase the accuracy (Daetwyler *et al.* 2010). This is provided that increasing the reference population is possible given, for example, the size of the actual population.

### Conclusions

The aim of this study was to investigate ways to reduce the loss of genetic diversity in GS breeding programs. The choice of individuals to be phenotyped and/or added to the reference population appeared to modestly impact the genetic gain and genetic diversity of the breeding population. The use of the OC strategy, taking into account both the relationships and performances of the individuals, to update the reference population: (i) allowed for better conservation of genetic diversity in the breeding population, (ii) predicted more accurate BV, and (iii) had only minor repercussions on the genetic gain. The results of this study support the use of the OC strategy as a way to update the reference population, especially for breeds in need of diversity conservation wanting to implement long-term GS programs. Making changes in the composition of the reference population impacted the breeding population characteristics and enabled the incorporation of genetic diversity in GS without revising farmers’ practices.

## Supplementary Material

Supplemental material is available online at www.g3journal.org/lookup/suppl/doi:10.1534/g3.117.1117/-/DC1.
