# Supplementary material for: Which Individuals To Choose To Update the Reference Population? Minimizing the Loss of Genetic Diversity in Animal Genomic Selection Programs
Source: G3 (Bethesda). 2017 Nov 13;8(1):113–21. doi: 10.1534/g3.117.1117 (PMC5765340; doi:10.1534/g3.117.1117)
Supplement: Supplementary file 1 [file 113FigureS1.pdf]

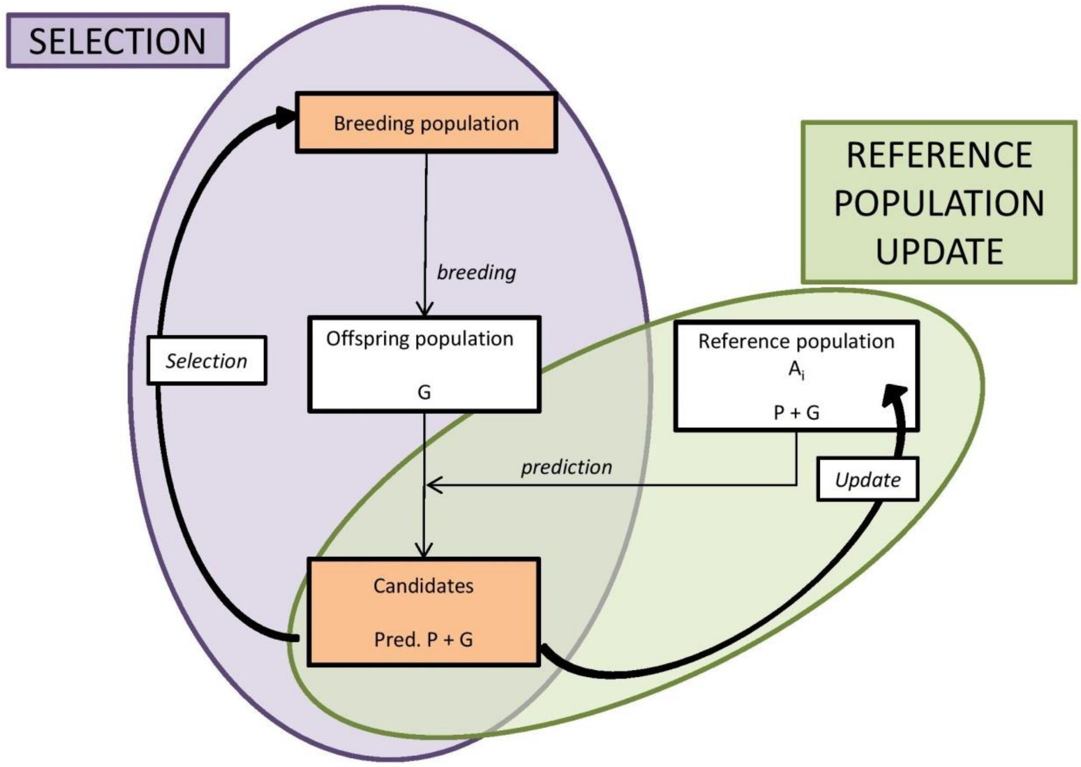

Figure S1 – Livestock breeding using Genomic Selection. This figure represents the main mechanisms of livestock breeding. The green circle is the representation of Reference Population update in Genomic Selection. The purple circle is the representation of the Selection scheme. P means phenotype, Pred. P means predicted phenotype and G means genotype, A_i is the reference population at the generation under scrutiny. The highlighted blocks represent the population of interest for the analysis.
