# Supplementary material for: Which Individuals To Choose To Update the Reference Population? Minimizing the Loss of Genetic Diversity in Animal Genomic Selection Programs
Source: G3 (Bethesda). 2017 Nov 13;8(1):113–21. doi: 10.1534/g3.117.1117 (PMC5765340; doi:10.1534/g3.117.1117)
Supplement: Supplementary file 2 [file 113FigureS2.pdf]

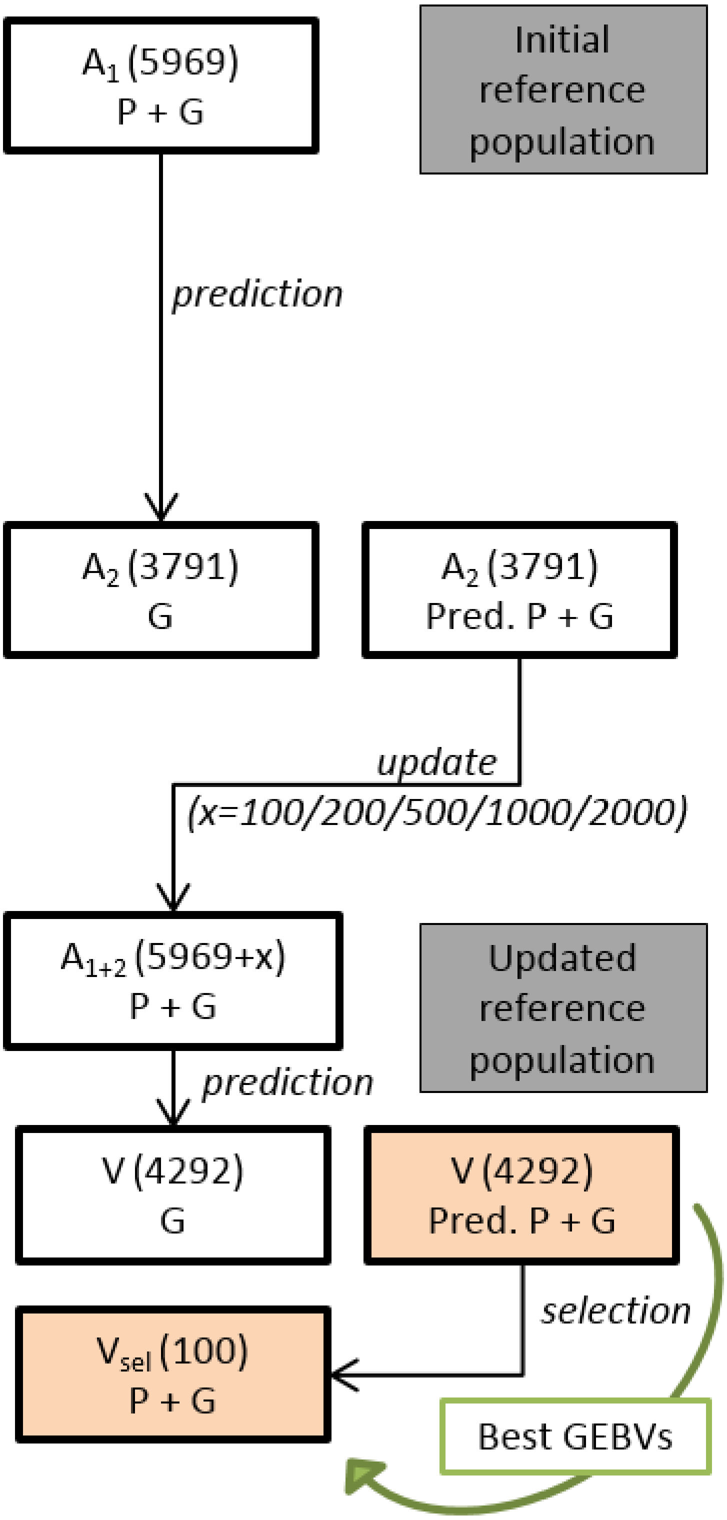

Figure S2- Detail of the analysis set up on the real dataset. P means phenotype, Pred. P means predicted phenotype and G means genotype, A_i is the reference population at the generation under scrutiny, V is the validation population and V_{sel} is the selected candidates for breeding in the next generation. The green arrows inform on the selection decisions either random or based on best EBVs. The highlighted blocks represent the populations of interest for the analysis.
