# Supplementary material for: Which Individuals To Choose To Update the Reference Population? Minimizing the Loss of Genetic Diversity in Animal Genomic Selection Programs
Source: G3 (Bethesda). 2017 Nov 13;8(1):113–21. doi: 10.1534/g3.117.1117 (PMC5765340; doi:10.1534/g3.117.1117)
Supplement: Supplementary file 3 [file 113FileS1.docx]

## Details of the simulation process

We aimed to simulate a population with similar characteristics as a domestic cattle population in order to infer the long term impact of reference population update on the breeding population genetic merit and genetic diversity. This was achieved by using an initial population of 700 males and 700 females that followed random mating for 255 generations with a slight decrease in population size, succeeded by 245 generation of imposing a drastic bottleneck, as observed *in natura*, to reach 100 individuals in total at generation 500. The simulated trait was assigned properties equal to empirical data for milk yield and genetic architecture was similar real data on bovine genome (Snelling *et al.* 2007). In the initial population each chromosome carried the same number of evenly spaced SNP markers as in the real dataset. To ensure a sufficiently large number of segregating QTL in the final data, 7,250 quantitative trait loci (QTLs) were randomly distributed across the genome, with QTL effects following a gamma distribution with a shape parameter of 0.4 (Meuwissen *et al.* 2001). Both SNPs and QTLs had equal allele frequencies in the initial population. The mutation rate of markers was set to 2.5*10^-5^.

The historical population was followed by 5 generations with increasing number of females; in generation 505 the population consisted of 25 males and 5,000 females. Followed 10 generations of breeding decisions based on Estimated Breeding Values (EBVs) estimated from a Best Linear Unbiased Prediction (BLUP) method. In the last of these generations 1,000 males and 1,000 females were randomly chosen as ancestral population of breeding individuals. In this population, on average, 39,572 SNP markers and 6,499 QTLs were still segregating. Ten more generations of selection and breeding were simulated, in every generation the 150 males and 500 females from the previous generation with highest GEBVs were selected to produce the next generation *n+1*. Each female produced one offspring per generation and the sex ratio in the offspring generation was 0.5. Simulations were performed using QMSim (Sargolzaei and Schenkel 2009).

References

Meuwissen, T. H. E., B. J. Hayes and M. E. Goddard, 2001 Prediction of total genetic value using genome-wide dense marker maps. Genetics 157**:** 1819-1829.

Sargolzaei, M., and F. S. Schenkel, 2009 QMSim: a large-scale genome simulator for livestock. Bioinformatics 25**:** 680-681.

Snelling, W. M., R. Chiu, J. E. Schein, M. Hobbs, C. A. Abbey *et al.*, 2007 A physical map of the bovine genome. Genome Biology 8**:** 17.


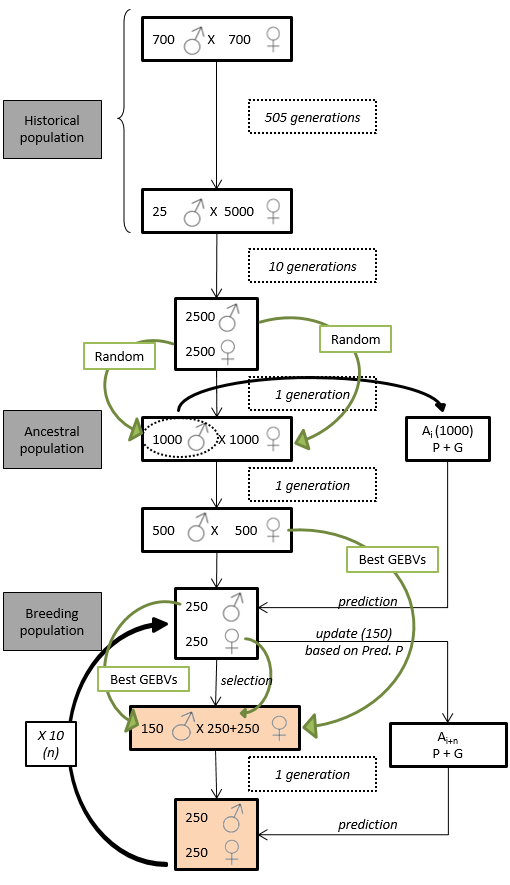
**Figure - Detail of the analysis set up on the simulated datasets.** P means phenotype, Pred. P means predicted phenotype and G means genotype, *Ai* is the reference population at the generation under scrutiny and *V* is the validation population. The green arrows inform on the selection decisions either random or based on best EBVs. The highlighted blocks represent the populations of interest for the analysis.
