# Supplementary material for: Which Individuals To Choose To Update the Reference Population? Minimizing the Loss of Genetic Diversity in Animal Genomic Selection Programs
Source: G3 (Bethesda). 2017 Nov 13;8(1):113–21. doi: 10.1534/g3.117.1117 (PMC5765340; doi:10.1534/g3.117.1117)
Supplement: Supplementary file 4 [file 113FileS2.docx]

# SUPPLEMENTARY TABLES

Table S1: **Variables and values considered for the three different strategies.**

| **Variables** | **Values taken** |
| --- | --- |
| **Real data set** |  |
| Selection strategies | *Random*, *Sel*, *SelDiv* |
| Selection criteria | Random, Truncation, Relationships, Genetic merit, Rate of inbreeding |
| Number of individuals added to reference population | 100 (+ 1.5 %), 200 (+ 3 %), 500 (+ 8 %), 1000 (+ 15 %), 2000 (+ 30 %) |
| Relationship matrix (*SelDiv*) | Similarity (S) |
| **Simulated data set** |  |
| Selection strategies | *Random*, *Sel*, *SelDiv* |
| Selection criteria | Random, Truncation, Relationships, Genetic merit, Rate of inbreeding |
| Number of individuals added to reference population | 150 |
| Relationship matrix (*SelDiv*) | Similarity (S) |

Table S2: **Descriptive statistics for the selected candidates for the different strategies and generations in the simulated data set.**

|  |  | **Breeding Value** | | **Absolute prediction bias** | | **Inbreeding** | | **Observed Heterozygosity** | |
| --- | --- | --- | --- | --- | --- | --- | --- | --- | --- |
| **Generation** | **Selection strategy** | **Average** | **95 % confidence interval** | **Average** | **95 % confidence interval** | **Average** | **95 % confidence interval** | **Average** | **95 % confidence interval** |
| **1** | ***Sel*** | 3657.3 | [3599.5 ; 3715.1] | 0.706 | [0.698 ; 0.714] | 0.115 | [0.111 ; 0.120] | 0.284 | [0.282 ; 0.286] |
|  | ***SelDiv*** | 3657.3 | [3599.5 ; 3715.1] | 0.709 | [0.700 ; 0.718] | 0.115 | [0.111 ; 0.120] | 0.284 | [0.282 ; 0.286] |
|  | ***Random*** | 3657.3 | [3599.5 ; 3715.1] | 0.710 | [0.703 ; 0.718] | 0.115 | [0.111 ; 0.120] | 0.284 | [0.282 ; 0.286] |
| **2** | ***Sel*** | 3857.4 | [3797.6 ; 3917.1] | 0.697 | [0.690 ; 0.704] | 0.117 | [0.112 ; 0.121] | 0.282 | [0.280 ; 0.284] |
|  | ***SelDiv*** | 3858.2 | [3798.1 ; 3918.4] | 0.699 | [0.690 ; 0.707] | 0.117 | [0.112 ; 0.121] | 0.282 | [0.280 ; 0.284] |
|  | ***Random*** | 3859.0 | [3799.4 ; 3918.6] | 0.706 | [0.697 ; 0.715] | 0.117 | [0.112 ; 0.121] | 0.282 | [0.280 ; 0.283] |
| **3** | ***Sel*** | 4050.1 | [3991.4 ; 4108.7] | 0.694 | [0.688 ; 0.701] | 0.118 | [0.114 ; 0.123] | 0.279 | [0.277 ; 0.281] |
|  | ***SelDiv*** | 4043.9 | [3982.7 ; 4105.2] | 0.693 | [0.686 ; 0.700] | 0.118 | [0.114 ; 0.122] | 0.280 | [0.277 ; 0.281] |
|  | ***Random*** | 4046.3 | [3985.9 ; 4106.7] | 0.704 | [0.696 ; 0.711] | 0.118 | [0.114 ; 0.123] | 0.279 | [0.277 ; 0.281] |
| **4** | ***Sel*** | 4244.6 | [4184.5 ; 4304.6] | 0.690 | [0.681 ; 0.699] | 0.119 | [0.115 ; 0.124] | 0.277 | [0.275 ; 0.279] |
|  | ***SelDiv*** | 4231.3 | [4168.9 ; 4293.8] | 0.693 | [0.686 ; 0.700] | 0.119 | [0.115 ; 0.123] | 0.278 | [0.276 ; 0.280] |
|  | ***Random*** | 4237.0 | [4176.6 ; 4297.5] | 0.715 | [0.705 ; 0.725] | 0.119 | [0.115 ; 0.124] | 0.277 | [0.274 ; 0.279] |
| **5** | ***Sel*** | 4437.2 | [4375.7 ; 4498.8] | 0.702 | [0.693 ; 0.711] | 0.120 | [0.116 ; 0.125] | 0.275 | [0.273 ; 0.277] |
|  | ***SelDiv*** | 4422.1 | [4357.2 ; 4487.0] | 0.692 | [0.685 ; 0.699] | 0.120 | [0.115 ; 0.124] | 0.276 | [0.274 ; 0.278] |
|  | ***Random*** | 4419.4 | [4356.1 ; 4482.7] | 0.706 | [0.698 ; 0.714] | 0.121 | [0.115 ; 0.125] | 0.274 | [0.272 ; 0.276] |
| **6** | ***Sel*** | 4621.7 | [4559.1 ; 4684.3] | 0.720 | [0.711 ; 0.728] | 0.121 | [0.117 ; 0.126] | 0.273 | [0.271 ; 0.276] |
|  | ***SelDiv*** | 4601.1 | [4535.0 ; 4667.1] | 0.710 | [0.701 ; 0.718] | 0.121 | [0.117 ; 0.126] | 0.274 | [0.271 ; 0.276] |
|  | ***Random*** | 4600.0 | [4535.2 ; 4664.8] | 0.737 | [0.720 ; 0.753] | 0.122 | [0.117 ; 0.126] | 0.272 | [0.270 ; 0.274] |
| **7** | ***Sel*** | 4809.1 | [4744.5 ; 4873.7] | 0.721 | [0.712 ; 0.729] | 0.123 | [0.118 ; 0.127] | 0.272 | [0.269 ; 0.274] |
|  | ***SelDiv*** | 4777.2 | [4708.5 ; 4845.9] | 0.706 | [0.698 ; 0.713] | 0.122 | [0.118 ; 0.127] | 0.272 | [0.270 ; 0.274] |
|  | ***Random*** | 4776.7 | [4710.7 ; 4842.7] | 0.739 | [0.725 ; 0.753] | 0.123 | [0.118 ; 0.127] | 0.270 | [0.268 ; 0.272] |
| **8** | ***Sel*** | 4990.0 | [4923.8 ; 5056.3] | 0.700 | [0.692 ; 0.709] | 0.124 | [0.119 ; 0.128] | 0.270 | [0.268 ; 0.272] |
|  | ***SelDiv*** | 4950.9 | [4880.8 ; 5020.9] | 0.690 | [0.682 ; 0.698] | 0.123 | [0.119 ; 0.128] | 0.270 | [0.268 ; 0.272] |
|  | ***Random*** | 4957.2 | [4890.3 ; 5024.2] | 0.719 | [0.708 ; 0.729] | 0.124 | [0.119 ; 0.128] | 0.268 | [0.266 ; 0.270] |
| **9** | ***Sel*** | 5174.8 | [5107.2 ; 5242.5] | 0.743 | [0.734 ; 0.752] | 0.125 | [0.120 ; 0.129] | 0.268 | [0.266 ; 0.270] |
|  | ***SelDiv*** | 5133.2 | [5060.6 ; 5205.8] | 0.728 | [0.718 ; 0.737] | 0.125 | [0.120 ; 0.129] | 0.268 | [0.266 ; 0.270] |
|  | ***Random*** | 5137.2 | [5067.7 ; 5206.7] | 0.770 | [0.755 ; 0.785] | 0.125 | [0.120 ; 0.129] | 0.266 | [0.264 ; 0.269] |
| **10** | ***Sel*** | 5357.0 | [5288.5 ; 5425.5] | 0.743 | [0.732 ; 0.754] | 0.126 | [0.122 ; 0.130] | 0.266 | [0.264 ; 0.268] |
|  | ***SelDiv*** | 5302.7 | [5228.0 ; 5377.4] | 0.735 | [0.726 ; 0.744] | 0.126 | [0.121 ; 0.130] | 0.267 | [0.265 ; 0.269] |
|  | ***Random*** | 5305.4 | [5235.8 ; 5375.0] | 0.808 | [0.792 ; 0.823] | 0.126 | [0.121 ; 0.130] | 0.265 | [0.262 ; 0.267] |

Table S3: **Summary of the linear models for each of the four variables analyzed in simulations.**

|  | **Sum of squares** | **Mean square** | **df** | **F** | **P-value (Chisq)** |
| --- | --- | --- | --- | --- | --- |
| **Breeding value** | | | | | |
| **Strategy** | 9.83*10^7^ | 4.9*10^7^ | 2 | 169.02 | 9.96*10^-6^ |
| **Generation** | 2.74*10^11^ | 2.74*10^11^ | 1 | 941 460.32 | < 10^-16^ |
| **Ne/N** | 1.61*10^8^ | 1.61*10^8^ | 1 | 554.66 | < 10^-16^ |
| **Strategy:Generation** | 6.35*10^7^ | 3.17*10^7^ | 2 | 109.17 | < 10^-16^ |
| **Prediction bias** | | | | | |
| **Strategy** | 675.60 | 337.80 | 2 | 432.82 | < 10^-16^ |
| **Generation** | 9379.70 | 9379.70 | 1 | 12 017.70 | < 10^-16^ |
| **Ne/N** | 34.10 | 34.10 | 1 | 43.63 | 1.52*10^-11^ |
| **Strategy:Generation** | 1163.20 | 581.60 | 2 | 745.15 | < 10^-16^ |
| **Inbreeding** | | | | | |
| **Strategy** | 1.24*10^-2^ | 6.20*10^-3^ | 2 | 12.17 | 3.52*10^-1^ |
| **Generation** | 27.80 | 27.80 | 1 | 54 414.35 | < 10^-16^ |
| **Ne/N** | 3.63*10^-1^ | 3.63*10^-1^ | 1 | 710.70 | < 10^-16^ |
| **Strategy:Generation** | 3.00*10^-3^ | 1.50*10^-3^ | 2 | 2.94 | 5.28*10^-2^ |
| **Observed heterozygosity** | | | | | |
| **Strategy** | 3.96*10^-1^ | 1.98*10^-1^ | 2 | 723.25 | 1.12*10^-2^ |
| **Generation** | 40.483 | 40.483 | 1 | 148 019.52 | < 10^-16^ |
| **Ne/N** | 7.00*10^-3^ | 7.00*10^-3^ | 1 | 24.40 | 1.26*10^-6^ |
| **Strategy:Generation** | 1.46*10^-1^ | 7.30*10^-2^ | 2 | 266.27 | < 10^-16^ |
